# Editorial: Heart Failure in Pediatrics and Congenital Heart Disease

**DOI:** 10.3389/fped.2022.936193

**Published:** 2022-06-17

**Authors:** Estela Azeka, Joshua Navarajasegaran, Giorgia Grutter, Dimpna Calila Albert-Brotons

**Affiliations:** ^1^Heart Institute (InCor) University of São Paulo Medical School, São Paulo, Brazil; ^2^Merton College, University of Oxford, Oxford, United Kingdom; ^3^Pediatric Hospital Bambino Gesu, Rome, Italy; ^4^Heart Center, King Faisal Specialist Hospital & Research Center, Riyadh, Saudi Arabia

**Keywords:** heart failure, pediatrics, congenital heart disease, ECMO, COVID-19, hybrid procedure, rehabilitation, cardio-oncology

Heart failure (HF) continues to contribute to the morbidity and mortality of children (and adults) with congenital heart disease (CHD). While significant strides have been made in the treatment of CHD and pediatric HF, there is still more to understand, learn, and implement in this area of pediatric medicine, especially when compared to our understanding and treatment of adult HF and its causes.

For far too long, as stressed in many of these articles, we have relied on and extrapolated from our knowledge of adult HF to treat the pediatric population. Given that, not only is this a vastly different population with different physiologies (even within the umbrella of pediatrics), but the initial etiologies leading to HF are distinct. With a wide range of areas studied, from the pathophysiology of HF in children to post-procedure complications, this Research Topic sought to bring together researchers from across the world with a variety of experiences to continue to enhance our knowledge in this area of pediatric cardiology in order to better equip clinicians to provide improved (holistic) care to those with CHD and/or HF.

There were 16 articles accepted for publication in this Research Topic including 109 authors, 9 original research projects, 1 brief research report, 1 mini review, 1 review, 1 systemic review, and 3 case reports.

## Heart Failure Pathophysiology

Fiegle et al. from Germany, reported the severe remodeling of the t-system, involving the loss of t-tubules and the remodeling to t-sheets, in children with acute myocarditis, from endomyocardial biopsies collected during implantation and explantation of ventricular assist devices. This study has begun to remove the current notion that t-system remodeling only occurs in chronic heart failure or ischaemic cardiomyopathy (predominantly in adults) but may contribute to the pathophysiology in acute HF driven by acute myocarditis. This study also suggests that further research on t-system recovery may be warranted as a potential therapeutic target.

Azeka et al. from São Paulo, reported a potential association between dilated cardiomyopathy (DCM) in a newborn possibly resulting from a SARS-CoV-2 infection, highlighting that, although rare, this could be a possible complication following COVID-19 infection.

## Surveillance/Prognostication

Tan C. et al. from China, studied the changes of the T-wave amplitude and QT interval between electrocardiograms (ECG) of children with DCM taken in supine and orthostatic positions. The results of this study introduce the idea that performing ECGs on children in these positions and analysis of the T-wave amplitude and QT interval difference could help not only diagnose DCM but also aid in prognosis and response to treatment, especially given the ease and availability of ECGs.

Kim et al. from Seoul, analyzed variables associated with the development of cardiac events (CE) and functional recovery (FR) in children with DCM. They proposed that a prediction model for pediatric DCM is a useful tool to prognosticate those patients who are more likely to suffer a CE and those more likely to have FR, and thus management strategies can be tailored accordingly.

Tan V. et al. from Singapore, reviewed the epidemiology of cardiotoxicity after anthracycline chemotherapy for childhood cancer in a multiethnic Asian population to tackle the lack of data in this population group. This retrospective study suggested that children with cardiotoxicity causing abnormal ventricular systolic function have a higher all-cause mortality risk compared to those with normal function and concluded that systematic echocardiogram surveillance was an important tool in monitoring those on anthracycline chemotherapy, given that a significant proportion of those patients with cardiotoxicity were asymptomatic.

Helle et al. from Finland, studied a cardiomyopathy gene panel to identify possible pathogenic variants associated with a poor prognosis in patients with hypoplastic left heart syndrome and investigated whether this would be a useful tool in clinical practice to help identify those at risk. The results from this study suggested that, at the current time, this is not a useful investigation.

Jang et al. from Minnesota, reported two cases of children with 1p36 deletion syndrome who presented with left ventricular non-compaction cardiomyopathy. These two cases added to the current literature of the importance of cardiac screening for children with this deletion syndrome, while also going a step further by highlighting positive outcomes in these cases through heart transplantation.

## Clinical Management

Wu Y. et al. from China, performed a systematic review and meta-analysis on the use extracorporeal membrane oxygenation (ECMO) after CHD repair to study the associated risk factors and outcomes. Amongst their findings, they concluded that there is still a high in-hospital mortality rate, but that single ventricular physiology and renal failure are independent risk factors that may contribute to the overall mortality. Bleeding was noted as a common complication. This paper noted that research is still somewhat limited in this field of extracorporeal life support and that further research, on ECMO and other such strategies, through the use of RCTs would be highly impactful and useful.

Lo et al. from Taiwan, presented a case report studying the use of a combination of angiotensin receptor inhibitor and neprilysin inhibitor in acute decompensated HF due to chemotherapy-induced cardiomyopathy. While this novel medication has been studied and approved in adults, this case gives insights into the possibility of its usage, appropriate dosage, and indication in the pediatric population. Here, in this patient it led to being an effective therapy when conventional medications were not.

Loss et al. from California, reviewed recently approved and upcoming drug therapies for pediatric HF. As briefly mentioned previously, pediatric HF therapies are understudied and difficult to develop in comparison to therapies for the adult population. This review gives us an insight into recently approved medications for children with HF (acute and chronic), and for adults, which may then eventually filter down for use in children, equipping us with more options for treatment in clinical practice. This review, however, stresses the importance of studying these drugs in children rather than extrapolating from the adult population.

Gil-Jaurena et al. from Madrid, reported their 8-year experience of hybrid procedures, where cardiac surgeons and cardiologists collaborate together to perform interventions, thereby expanding the possible treatment options that would normally be available to one specialty working alone. Although not without complications, this report showed the success and importance of collaboration between cardiac surgeons and cardiologists; where previous endeavors and expectations can be broken, and new possibilities are plenty.

Lenoir et al. from France, compared the short- and medium-term mortality between two alternate techniques to tackle patent ductus arteriosus (PDA) in very low-weight (<1,600 g) preterm infants: minimally invasive surgical PDA ligation by anterior minithoracotomy and transcatheter PDA closure. They concluded that not only is there equivalent efficiency between these two procedures but also comparable levels of safety. Thus, Lenoir et al. proposed that the choice of procedure to treat PDAs in this population should be driven by the center's facilities and competences of those delivering the therapy.

Tikkanen et al. from Harvard Medical School, discussed the importance of rehabilitation interventions in the pediatric HF population to tackle the impact of functional status caused by the condition itself, its complications or treatments, including cardiac transplant. They positively noted the improvements in therapies and treatments to tackle HF in children leading to increasing survival, however, stressed that much more needs to be done to improve the quality of life that they are left with. This review calls for an individualized multi-disciplinary approach to rehabilitation for children with HF, including areas such as speech and swallow, to support these children to live with an improved function and quality of life.

Werninger et al. from Switzerland, investigated social and behavioral outcomes in 10-year-old children with congenital heart disease (CHD). Whilst no differences in general behavioral difficulties were seen compared to normative data, the team noted an increase in attention-deficit-hyperactivity-disorder related symptoms and social interaction problems. Furthermore, this paper also showed that IQ and maternal mental health at age 4 were predictive for these outcomes, thus highlighting the importance of supporting the parents, especially those with mental health issues, of children with CHDs.

## Post-Procedure Complications

Wu Z. et al. from China, reviewed the clinical characteristics, prognosis, and related risk factors of left anterior fascicular block (LAFB) after transcatheter closure of ventricular septal defects (VSDs) in children; a common congenital heart defect. They noted whilst LAFB is not an uncommon side effect after this procedure, the prognosis was encouraging as no progressive severity was found that influences poorly on cardiac function, such as complete bundle branch block.

Yang et al. from China, reported the causes and risk factors of unplanned surgery after transcatheter closure of VSDs in children. Again, this study looked to fill in a gap regarding complications of post-transcatheter closure of VSDprocedures. It was concluded that new-onset or worsening aortic regurgitation was the most common cause of needing an unplanned surgery post procedure, while various cardiac function markers, such as pulmonary artery systolic pressure ≥ 45 mmHg, and anatomical features of the child's heart, including a primary aortic valve prolapse or intracristal VSD, would increase the risk of returning to theater.

Overall, we have gained more insight into the pathophysiology of HF in children, possible ways to implement surveillance to earlier identify patients, better models to prognosticate patients, as well as useful research to help answer a range of management questions, be it regarding therapeutic medications or therapeutic interventions. Altogether, every bit of basic to clinical research will aid us in our continued ambition to better serve this population with CHD and HF.

There are still many issues in heart failure and congenital heart disease which are unknown where the future research should focus on like pharmacogenomics in heart failure, intervention in congenital heart disease in fetus as well as improve the quality of life in this population.

## Author Contributions

EA prepared and proofed the manuscript. JN, GG, and DA-B prepared and revised the manuscript. All authors approved the submitted version.

## Conflict of Interest

The authors declare that the research was conducted in the absence of any commercial or financial relationships that could be construed as a potential conflict of interest.

## Publisher's Note

All claims expressed in this article are solely those of the authors and do not necessarily represent those of their affiliated organizations, or those of the publisher, the editors and the reviewers. Any product that may be evaluated in this article, or claim that may be made by its manufacturer, is not guaranteed or endorsed by the publisher.

